# Surgical Treatment of Trigeminal Neuralgia1An address read before the Tristate Medical Association of the Carolinas and Virginia, at White Stone Lithia Springs, S. C., February 27 and 28, 1906.

**Published:** 1906-04

**Authors:** Benjamin Merrill Ricketts

**Affiliations:** Cincinnati


### Surgical Treatment of Trigeminal Neuralgia[1]

By BENJAMIN MERRILL RICKETTS, M. D., Cincinnati.

*(Author's Abstract.)*

TRIFACIAL neuralgia is one of the most desperate conditions that does not terminate in death that the surgeon is called upon to relieve. Its etiology and pathology remain unknown but the change in posture from bipedal to quadrupedal is probably a factor in its causation. The technic of each operation is given in detail together with the bibliography of

1. Neurectomy and neurotomy.
2. Ligation of carotid arteries.
3. Removal of Gasserian ganglion.

Neurotomy, neurectomy, and the injection of osmic acid accomplish the same purpose, giving relief, but for a short time, when regeneration will be established. Thiersch says that regeneration will occur after 5 cm have been removed. The effect of osmic acid injected into the nerve is the same as constricting the nerve with a ligature. The nerve tissue becomes hardened but regeneration will occur.

Avulsion as practised by LaPlace absolutely prevents regeneration. The nerve is divided as it emerges from its foramen, the body grasped with forceps and twisted slowly from right to left and from left to right until the body, together with its filaments, is removed.

Lexer's method (a modification of Kronlein's) is as follows:

1. Cut flap within lines indicated.
2. Divide zygoma with saw at each extremity.
3. Cut away the malar arch with the chisel.
4. Retract the flap and remove the zygoma.
5. Detach the soft tissues to the infraorbital crest.
6. Separate the original or the external pterygoid.
7. The base of the skull and the foramen ovale are now exposed.

---

1. An address read before the Tristate Medical Association of the Carolinas and Virginia, at White Stone Lithia Springs, S. C., February 27 and 28, 1906.

8. Divide the branch at its exit.
9. Ligate the middle meningeal artery and vein.
10. Tampon for any bleeding from pterygoid venous plexus.
11. Remove the second and first branches also if necessary.

After division of the nerves at their point of exit, avulsion is practised similar to the method of LaPlace. Lexer and Von Hook each report several cases done in this way.

The inferior dental nerve may be removed by incising the soft tissues over the inferior maxillary, retracting the parts and drilling to the posterior dental foramen. If necessary, the jaw may be channeled as far forward as the anterior dental foramen. The nerve may be avulsed without channeling; after dividing it as it enters and makes its exit from the jaw, by grasping it with a pair of forceps which have been passed through the opening at the posterior dental foramen.

Neurectomy as practised by Langenbeck is one of the most effectual methods because, next to Lexer's, it entails the destruction of more nerve tissue. It has long been practised. only by a few, however, owing to the want of skill and courage. It requires more skill than even the method of Lexer.

Ligation of the common and external carotid arteries above the occipital and facial branches for trifacial neuralgia has been done by Hutchinson, 1885, Fowler, 1896, and Ricketts, 1896, resulting in much relief from pain. It is probably an operation that should be combined with neurotomy, neurectomy, injection of osmic acid, or the partial removal of the ganglion. Gross, 1885, combined it with neurotomy.

The nerves originating in the ganglion of Gasser being both sensory and motor, are then rendered doubly difficult to deal with. The total destruction of the ganglion results in the loss of sensation and more or less motion, some of which is exceedingly undesirable. Complete removal of the ganglion probably results in greater mortality than partial removal, and the loss of sensation is proportionate to the amount of its destruction. Mortality in either complete or incomplete removal of the ganglion is influenced by the amount of time, trauma, anesthetic, hemorrhage and shock.

Horsley, 1891, Rose-Andrews, 1892, Hartley-Krause, 1892, Doyen, 1894, Cushing, 1900, and Kronlein, 1904, have each given technic for the removal of the ganglion that give immunity and mortality varying in degree. Preference has been given to the Hartley-Krause method, but even this has given an unreasonable mortality—namely 25 to 30 per cent. This alone should be sufficient cause for its discontinuance.

Kronlein's method is the second choice, having given less mortality than the Hartley-Krause operation. However, all intracranical operations devised for the removal of the Gasserian ganglion necessitate so much trauma to the smaller nerves not associated with pain from the fifth,. that destruction of the ganglion should be discontinued until methods with less trauma and mortality can be devised.

Kronlein's intracranial method is a continuation of the Lexer modification after the skull and foramen ovale have been exposed, the following being the several steps:

1. . Skull opened by chisel or drill.
2. Enlarge opening to foramen ovale with forceps.
3. Introduce finger to dura.
4. Push dura to foramen ovale.
5. Third nerve now seen inside the skull.
6. Raise body to a semisitting posture.
7. Wait until cerebral fluid gravitates into spinal canal.
8. Dissect away ganglion.

The unpleasant consequences possible to follow the operation may be grouped thus:

1. Injury to the motor root of the fifth nerve, paralyzing the muscles of mastication.

2. Paralysis of the buccinator, while never complete, results in more or less anchylosis due to (Quincke) trophic changes and contraction of the temporal and masseter muscles.

3. Complete corneal and conjunctival anesthesia, resulting in ulceration, opacity and softening of the cornea. (Cause not known, but trauma and infection suspected). Injury to the superficial petrosal nerve also suspected.

4. Ptosis, which is due to injury of the third, fourth and sixth nerves.

5. More or less loss of hearing resulting from paralysis of the tensor tympani, due to injury to the motor root of the fifth or to edema of tympanic membrane.

CONCLUSIONS

1. Avulsion of the distral branches should be the first operation resorted to.

2. Avulsion with ligation of common and external carotid arteries should be second choice.

3. Removal of the branches of the nerve (Lexer) should be the third choice.

4. Lexer's method combined with that of LaPlace, the fourth.

5. Lexer's method, combined with that of LaPlace and ligation of the common and external carotid arteries, the fifth.

6. Removal of the ganglion and neurectomy of the distal branches, the sixth.

7. Removal of the ganglion, combined with neurectomy and ligation of the common and external carotid arteries, the seventh.

8. Neurotomy, neurectomy or the injection of osmic acid gives only temporary relief.

9. Plugging the foramenæ with fragments of bone cut from the neighboring plate will prevent regeneration of the nerves passing through them.

10. The method of Kronlein is an innovation, having given better results with less deformity, mortality, loss of time, motor paralysis and less risk of loss of vision.

11. Relapse occasionally occurs after intracranial operations, but in such cases removal of the ganglion is supposed to have been incomplete.

12. All intracranial operations for the removal of the ganglion should be abandoned because of the high mortality, if for no other reason.

408 BROADWAY.

